# Molecular Profile of Variants in *CDH1*, *TP53*, *PSCA*, *PRKAA1*, and *TTN* Genes Related to Gastric Cancer Susceptibility in Amazonian Indigenous Populations

**DOI:** 10.3390/jpm13091364

**Published:** 2023-09-08

**Authors:** Kaio Evandro Cardoso Aguiar, Izabela De Sousa Oliveira, Amanda De Nazaré Cohen-Paes, Rita De Cássia Calderaro Coelho, Lui Wallacy Morikawa Souza Vinagre, Juliana Carla Gomes Rodrigues, André Maurício Ribeiro-Dos-Santos, Sandro José De Souza, Ândrea Ribeiro-Dos-Santos, João Farias Guerreiro, Paulo Pimentel de Assumpção, Sidney Emanuel Batista Dos Santos, Ney Pereira Carneiro Dos Santos, Marianne Rodrigues Fernandes

**Affiliations:** 1 Oncology Research Center, Federal University of Pará, Belém 66073-005, PA, Brazil; kaio.evandro@hotmail.com (K.E.C.A.); julianarodrigues@gmail.com (J.C.G.R.); npcsantos.ufpa@gmail.com (N.P.C.D.S.); 2 Laboratory of Human and Medical Genetics, Institute of Biological Science, Federal University of Pará, Belém 66077-830, PA, Brazil; 3 Brain Institute, Universidade Federal do Rio Grande do Norte, Natal 59078-970, RN, Brazil

**Keywords:** gastric cancer, genomics, exome, indigenous population

## Abstract

Gastric Cancer is a disease associated with environmental and genetic changes, becoming one of the most prevalent cancers around the world and with a high incidence in Brazil. However, despite being a highly studied neoplastic type, few efforts are aimed at populations with a unique background and genetic profile, such as the indigenous peoples of the Brazilian Amazon. Our study characterized the molecular profile of five genes associated with the risk of developing gastric cancer by sequencing the complete exome of 64 indigenous individuals belonging to 12 different indigenous populations in the Amazon. The analysis of the five genes found a total of 207 variants, of which 15 are new in our indigenous population, and among these are two with predicted high impact, present in the *TTN* and *CDH1* genes. In addition, at least 20 variants showed a significant difference in the indigenous population in comparison with other world populations, and three are already associatively related to some type of cancer. Our study reaffirms the unique genetic profile of the indigenous population of the Brazilian Amazon and allows us to contribute to the conception of early diagnosis of complex diseases such as cancer, improving the quality of life of individuals potentially suffering from the disease.

## 1. Introduction

Gastric Cancer is a multifactorial disease associated with genetic alterations or environmental questions. It is the fifth most common cancer and fourth in cause of death worldwide, with a high incidence in East Asia and Latin America [1]. In the northern region of Brazil, Gastric Cancer is the second most frequent cancer in men and fifth in women, according to data released by the National Institute of Cancer (2023) [2].

This scenario may have influenced the ancestral genetic profile of this population. Studies demonstrate that genomic ancestry has a great influence on the clinical presentation and the incidence of Gastric Cancer in different populations according to their historical formation [3,4]. The Brazilian population is one of the most mixed around the world, with the important contribution of three ancestral populations: Europeans, Africans and Indigenous people. This mixing directly impacts the fluctuation of genetic variants’ frequency that may act on predisposition to diseases with clinical manifestations, such as cancer [5]. It is known that the mixed Brazilian population presents approximately 30% of the indigenous genomic contribution; therefore, studies in this ancestral population potentially assist in the reduction of problematic Gastric Cancer in the country [6,7].

Despite being a highly type of neoplastic study, only rare studies have been conducted in populations genetically heterogeneous or in original people, and an even smaller portion of the Brazilian indigenous population [8]. In this way, it is extremely valid to analyze the genetic profile of this original population of the Brazilian Amazon, in order to investigate genetic variants already elucidated in the world literature, as well as unknown or unscreened variants that may be associated with the development of Gastric Cancer.

Many studies demonstrate that the single nucleotide variants (SNVs) in target genes can assist in the carcinogenesis process [9,10]. Especially for Gastric Cancer, the important role of genes related to metabolic pathways, accession, proliferation, and cellular survival could be highlighted by intense participation in tumor development [11,12]. Thus, the objective of this study was to investigate the exome of genes *TP53*, *CDH1*, *PSCA*, *PRKAA1* and *TTN* in the indigenous population of the Brazilian Amazon and characterize the variants that may be associated with the risk of Gastric Cancer in this population.

## 2. Materials and Methods

### 2.1. Population Analysis for the Sutdy

The study is composed of 64 indigenous people from the Amazon in the northern region of Brazil, represented by 12 different indigenous peoples: Asurini located in Xingu and Tocantins, Arara, Araweté, Awa-Guajá, Juruna, Kayapó/Xikrin, Karipuna, Munduruku, Phurere, Wajãpi and Zo’é. All participating individuals were grouped into a single group called Indigenous (INDG) for statistical analyses. Genetic ancestry data were obtained from a panel with 64 informative ancestry markers (IAM), as described by Ramos et al. All participants or their community leaders were instructed about the research to be carried out and signed an Informed Consent Form (TCLE). The study was approved by the National Research Ethics Committee (CONEP) and by the Research Ethics Committee of the Tropical Medicine Center of the Federal University of Pará (CAE: 20654313.6.0000.5172).

The population frequencies of indigenous people were compared with other continental populations: Europe (EUR), Africa (AFR), East Asia (EAS), South Asia (SAS) and Americas (AMR), present in the 1000 Genomes Database, version 3 (available at: http://www.1000genomes.org; accessed on 15 May 2023). The study included 503 subjects from Europe, 661 from Africa, 504 from East Asia, 489 from South Asia and 347 from the Americas.

### 2.2. DNA Extraction and Exome Analysis

DNA extraction was performed according to the PhenolChloroform method [13] with modifications. The quantification of the extraction product was performed by Nanodrop-8000 spectrophotometer (Thermo Fisher Scientific Inc., Wilmington, DE, USA) and the prospective analysis of the quality of the extracted material was performed using 2% agarose gel electrophoresis.

The variant library (exome) was prepared using Nextera Rapid Capture Exome (Illumina^®^, San Diego, CA, USA) and SureSelect Human All Exon V6 (Agilent Technologies, Santa Clara, CA, USA), following the kit protocol provided by the manufacturer. The sequencing reaction was performed by NextSeq 500^®^ platform (Illumina^®^, San Diego, CA, USA) using the NextSeq 500 High-output v2 Kit 300 cycle kit (Illumina^®^, San Diego, CA, USA).

### 2.3. Selection of Genes

The selection of genes was carried out by consulting the Pubmed database (pubmed.ncbi.nlm.nih.gov; accessed on 21 May 2023). The five selected genes (*TP53*, *CDH1*, *PSCA*, *PRKAA1* and *TTN*) are among the most commonly cited in the literature related to susceptibility to Gastric Cancer.

### 2.4. Selection of Variants

The selection was the result of using two evaluation criteria. First, a minimum of 10 coverage readings was carried out for each variant presented in subjects. Second, the impact of variants was studied, considering only those with high risk, moderate or modifier effect, according to the classification by SNPeff (https://pcingola.github.io/SnpEff/; accessed on 25 May 2023). As a result of the exome analysis, 307 variants were found, as shown in Supplementary Table S1. After the selection based on the criteria mentioned above, a total of 237 variants remained to be followed in the investigation process.

### 2.5. Statiscal and Bioinformatics Analysis

The study population had its allele frequency obtained by calculating genes and comparing with other large populations already investigated (EUR, AMR, EAS, SAS and AFR). For the evaluation of statistical significance in the differentiation of frequencies between populations, Fisher’s exact test was used. Population variability of polymorphisms was observed using Wright’s fixation index (FST). A *p*-value of ≤0.05 was considered as significant data. All investigation was performed in RStudio v.3.5.1.

Bioinformatics analyses were performed as previously described by Cohen-Paes et al., 2022 [14].

## 3. Results

**Figure 1:**
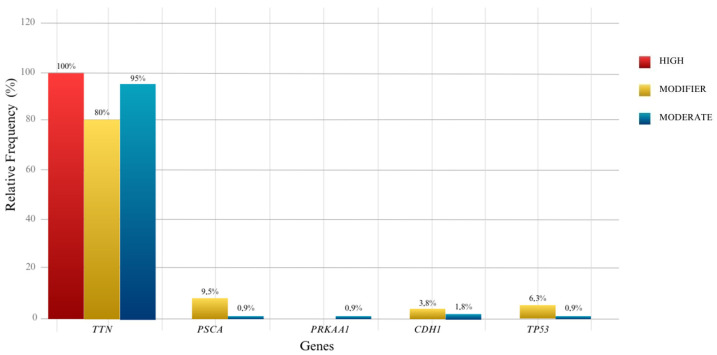
The relative contribution of variants discriminates according to the high, modifier and moderate impact in the *TTN*, *PSCA*, *PRKAA1*, *CDH1* and *TP53* genes.

Of 237 variants evaluated after the selection process, 207 belong to the TTN gene, one to the PRKAA1 gene, 13 to the PSCA gene, seven to the CDH1 gene and nine to the TP53 gene. Of these, it was possible to verify that the three variants were predicted with high impact, 126 modifiers and 108 moderates. From these analyses, the graphic of relative frequency (Figure 1) demonstrates the variance of the impact between the five genes studied, in which the significance of the TTN gene was visibly higher when compared to the other genes.

Quantitatively, 100% of the three variants with high impact are present in the *TTN* gene, two being new in the indigenous population. Furthermore, 80% presented modifier impact corresponding to 101 variants and 95% with moderate impact, equal to 103 variants. The *PSCA*, *PRKAA1* and *TP53* genes have the same 0.9% frequency with moderate impact, equal to 1 variant each; the *CDH1* gene has two variants with a frequency of 1.8% of the same moderate impact. With modifier impact, the *PSCA* gene has 9.5% corresponding to 12 variants, the *CDH1* gene t3.8% with 5 variants, and the *TP53* gene 6.3% with 8 variants. In the *PRKAA1* gene, no modifier variant was identified.

Table 1 describes the characteristics of the variants predicted by the software SNPeff with high and modifier impact, including the affected gene, the Id reference, chromosomic region, clinical impact and the frequency of alleles referent to the Indigenous population and the five great populations described by the 1000 genomes database (AFR, AMR, EAS, EUR and SAS). The variants with moderate impact are described in Supplementary Table S1.

Among the variants described in Table 1, the rs556408709 of the *TTN* gene has a high impact, characterized by a nucleotide change from C to T. Among the high impact variants, two are new variants also located on chromosome 2, the first at position 178597932 characterized by an Insertion/Deletion causing a change in the reading matrix, and the second at position 178651537, also an Insertion/Deletion changing the splice site acceptor. In addition, another 15 new variants are exclusive to the Indigenous population, 14 in the *TTN* gene and one in the *CDH1* gene (Table 2).

**Figure 2:**
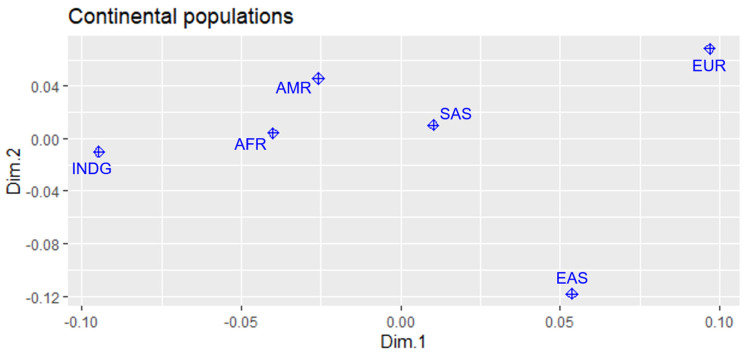
Multidimensional scaling plot illustrating Indigenous population and continental population according to genetic traits of variants in the *TTN*, *PSCA*, *PRKAA1*, *CDH1* and *TP53* genes.

The graph of the Multidimensional Scale Analysis (MDS) using the FST values and the genotypes of the populations for comparison between the 237 variants in the genes, shows a division into three fields (Figure 2).

It is possible to observe the genotypic distance of the Indigenous population compared with the other populations. Considering only the exome of the genes in this study, it can be concluded that the indigenous population is genetically closer to Americans and more distant from Europeans, which shows field isolation.

## 4. Discussion

Gastric cancer is one of the leading causes of death in Latin America. Several investigations regarding susceptibility to Gastric Cancer have been developed, with Whole Exome Sequence Analysis being one of the most promising methods. However, for the more than 1 million indigenous people living in the Brazilian territory according to the Brazilian Institute of Geography and Statics (IBGE), little attention has been paid to understanding the high incidence of cancer cases in this population [15].

The determination of the people originating from the Brazilian Amazon as an object of sequencing study derives from the manifestation of a unique genetic profile observed in this group, when compared with other world populations. It is known that this differentiation was due, in part, to the past colonization process that directly influenced the genetic variability of these peoples, implying the fluctuation of polymorphisms that influence the predisposition to different diseases [16], including Gastric Cancer. Thus, this research aimed to understand the genomic profile of the indigenous population (INDG) by evaluating five genes associated with gastric cancer applied to 12 different indigenous communities.

Our results described 237 variants never before described in these populations, at least five of which stood out due to their high clinical impact, at least five variants standing out for their predicted high clinical impact or statistical significance. The rs556408709 variant in the *TTN* gene, although it does not have a great difference in terms of frequencies when compared to other world populations, has high clinical relevance according to ClinVar (NCBI) with a prognosis not yet reported. The rs397517782 and rs397517532 variants, also in the TTN gene, were statistically significant in the INDG population when compared to AFR, AMR, EUR and SAS. *TTN* is responsible for encoding a transmembrane protein present in striated muscle tissues, in addition to being studied in different populations, being correlated with poor prognosis for Gastric Cancer in Chinese, and the most prominently related to various cancers, found in about 56% of tumors [17,18,19]. Variants in specific genes, such as *TTN*, can influence cell proliferation that leads to cancer, in which studies that investigated the accumulation of mutations in several genes that lead to gastric adenocarcinoma showed *TTN* among the 10 most mutated genes [20].

Additionally, rs1625895, present in *TP53*, showed statistical significance compared with the EUR and SAS populations. This intronic variant is associated with certain types of cancer such as lung, colorectal and ovarian cancer, in addition to the significant gene–environment interaction with a predisposition to oral cancer [21,22]. The *TP53* gene, which encodes the p53 tumor suppressor protein responsible for regulating the cell cycle, repair mechanisms and cell apoptosis from a transcriptional factor, is often described in cases of cancer as being essential for regulation against the development of neoplasms [23]. The presence of germline genetic variants in the *TP53* gene may influence the clinical presentation of diseases such as the Li–Fraumeni syndrome, characterized by a predisposition to multiple types of autosomal dominant cancer of early onset [24,25]. Other studies have sought to describe the genetic variability in the TP53 gene applied to other Brazilian indigenous peoples other than our study population, finding possible associations with the cancer progression process [26].

In addition, an important and expressive result in our analyses was the 17 new variants found in the indigenous population, and therefore possibly exclusive to this population. Among these, four are of the INDEL type, two of which are expected to have a high clinical impact. A significant portion of 16 new variants were found in the *TTN* gene, including the two high-impact variants, and the other new variant, the INDEL type, was found in the *CDH1* gene, which is highly associated with the development of Gastric Cancer. This tumor suppressor gene located on chromosome 16q22.1, which encodes the E-cadherin glycoprotein, is associated with cell adhesion for the formation of complex tissues associated with other proteins, in which mutations in the *CDH1* gene with loss of function of its protein can lead to cell–cell adhesion instability and important signaling failure in the pathways in which they act [11]. More than 100 pathogenic germline variants have already been described in the *CDH1* gene and studies, such as that by Luo and collaborators (2018) [27], demonstrate the relevance of the molecular mechanisms of carriers of mutations in the *CDH1* gene for diagnosis and treatment. Therefore, since *CDH1* is a Gastric Cancer target gene, we can state that the new variant found in our investigation may have an extremely important role in terms of cell cycle modulation, making it necessary to carry out further investigations to understand its biological impact.

The multidimensional scale chart presented in this study has characteristics that differentiate it from other analyses that follow the same standard methodology. This difference occurs mainly when we analyze the genetic proximity of the INDG population regarding the AFR population when compared with the other populations analyzed in the study. The AMR population also has high similarity, which is expected, due to the genetic contribution of the INDG population to the historical formation of mixed American peoples.

Finally, with the analysis of the results, the need is understood for studies like these that seek the genomic understanding of indigenous communities, for a better understanding of mechanisms of susceptibility and progression of gastric cancer. Thus, our findings contribute to knowledge of the genetic profile of Indigenous populations and may help in the development of new case-control studies that assess the clinical impact of scientifically elucidated variants and variants exclusive to Indigenous populations of the Amazon and, in addition, to corroborate studies carried out in mixed races of the Brazilian population.

## 5. Conclusions

This study evaluated the presence of genetic variants in five genes associated with Gastric Cancer susceptibility in different indigenous groups in the Brazilian Amazon. Our results demonstrate the presence of genetic variants in well elucidated genes and the identification of a large number of variants, including the discovery of new ones in other genes, such as TTN, showing the importance of studies applied to the indigenous population.

The findings in our study allow us to reaffirm the unique genetic profile of the indigenous population of the Amazon and mixed races in northern Brazil. Our data can contribute to the design of markers that help in the early diagnosis of complex diseases such as cancer, improving the quality of life of individuals potentially susceptible to the disease. In addition, our study can be a starting point for a better understanding of the particularities of this population.

## Figures and Tables

**Table 1:** Descriptions of the variants in the *TTN*, *PSCA*, *PRKAA1*, *CDH1* and *TP53* genes according to high impact and modifier, in addition to continental populations (African (AFR), American population (AMR), East Asian (EAS), European (EUR), and South Asian (SAS)) described in the 1000 genomes database.

Gene	Chromossome	Position	Reference	Variant	SNP Id	Impact	Var Type	INDG *	AFR *	AMR *	EAS *	EUR *	SAS *
*TTN*	Chr2	178704146	C	T	rs556408709	High	Snv	0.000	-	0.001	-	-	-
*TTN*	Chr2	178634311	A	G	rs7590037	Modifier	Snv	0.392	0.157	0.156	0.024	0.033	0.180
*TTN*	Chr2	178745892	C	G	rs16866489	Modifier	Snv	0.019	0.020	0.003	-	-	-
*TTN*	Chr2	178685342	T	C	rs16866434	Modifier	Snv	0.000	0.033	0.017	0.012	0.013	0.017
*TTN*	Chr2	178722960	T	C	rs2742327	Modifier	Snv	0.568	0.612	0.399	0.659	0.225	0.474
*TTN*	Chr2	178536584	C	CA	rs397517782	Modifier	Indel	0.100	0.004	0.006	-	0.008	0.005
*TTN*	Chr2	178717878	A	G	rs6750145	Modifier	Snv	0.028	0.298	0.196	0.436	0.160	0.274
*TTN*	Chr2	178669342	G	A	rs181914612	Modifier	Snv	0.000	0.003	0.003	-	-	-
*TTN*	Chr2	178730861	TA	T	rs376726021	Modifier	Indel	0.083	-	0.026	0.002	0.019	0.004
*TTN*	Chr2	178678713	A	C	rs184108456	Modifier	Snv	0.000	-	0.012	-	-	-
*TTN*	Chr2	178783080	AAAT	A	rs752207098	Modifier	Indel	0.000	-	-	-	-	-
*TTN*	Chr2	178748420	G	C	rs75785339	Modifier	Snv	0.017	0.141	0.010	-	-	-
*TTN*	Chr2	178592719	A	G	rs72646841	Modifier	Snv	0.000	0.135	0.050	0.180	0.045	0.063
*TTN*	Chr2	178546938	A	G	rs2288327	Modifier	Snv	0.014	0.289	0.199	0.630	0.172	0.319
*‘TTN*	Chr2	178730850	GT	GTT	rs35147775	Modifier	Indel	0.000	-	-	-	-	-
*TTN*	Chr2	142682339	T	G	rs10580462	Modifier	Indel	0.000	-	-	-	-	-
*TTN*	Chr2	178730850	GT	G	rs35147775	Modifier	Indel	0.000	-	-	-	-	-
*TTN*	Chr2	178730390	C	G	rs17076	Modifier	Snv	0.065	0.286	0.339	0.669	0.189	0.277
*TTN*	Chr2	178746176	A	G	rs529571649	Modifier	Snv	0.000	-	-	-	0.001	-
*TTN*	Chr2	178681069	A	G	rs72650039	Modifier	Snv	0.083	0.003	0.003	-	-	-
*TTN*	Chr2	178766637	AC	A	rs140501763	Modifier	Indel	0.426	-	-	-	-	-
*TTN*	Chr2	178702075	T	TA	rs397517532	Modifier	Indel	0.000	0.222	0.159	0.028	0.038	0.186
*TTN*	Chr2	178728858	T	C	rs16866476	Modifier	Snv	0.000	0.020	0.003	-	-	-
*TTN*	Chr2	178748531	A	C	rs72648906	Modifier	Snv	0.000	0.076	0.004	-	-	-
*TTN*	Chr2	178783097	G	A	rs72647864	Modifier	Snv	0.025	-	0.053	-	0.032	0.022
*TTN*	Chr2	178689003	ATTT TTTT	A	rs1242467782	Modifier	Indel	0.250	0.284	0.029	-	0.038	0.122
*TTN*	Chr2	178653350	A	G	rs13420457	Modifier	Snv	0.083	-	-	-	-	-
*TTN*	Chr2	178779207	A	G	rs2291301	Modifier	Snv	0.930	0.151	0.050	0.117	0.008	0.041
*TTN*	Chr2	178689190	G	A	rs2627038	Modifier	Snv	0.419	0.509	0.231	0.129	0.076	0.277
*TTN*	Chr2	178688666	C	T	rs369265969	Modifier	Snv	0.000	-	-	-	-	-
*TTN*	Chr2	178752043	GAA	G	rs397517802	Modifier	Indel	0.000	-	-	-	-	-
*TTN*	Chr2	178769662	TA	T	rs570467105	Modifier	Indel	0.051	0.008	0.001	0.006	-	-
*TTN*	Chr2	178804500	CTGGAG	C	rs3830329	Modifier	Indel	0.106	0.090	0.045	0.117	0.009	0.039
*TTN*	Chr2	178647040	GTATA	G	rs1459735441	Modifier	Indel	0.000	-	-	-	-	-
*TTN*	Chr2	178535858	GA	G	rs749872538	Modifier	Indel	0.146	0.003	0.002	0.001	0.001	0.002
*TTN*	Chr2	178789586	A	G	rs13421990	Modifier	Snv	0.179	0.012	0.150	0.016	0.035	0.162
*TTN*	Chr2	178779159	A	G	rs2291302	Modifier	Snv	0.930	0.151	0.050	0.117	0.008	0.041
*TTN*	Chr2	178706970	C	T	rs10203085	Modifier	Snv	0.016	0.102	0.147	0.026	0.036	0.177
*TTN*	Chr2	178750225	A	G	rs10803917	Modifier	Snv	0.984	0,093	0,003	-	-	-
*TTN*	Chr2	178654184	TAGC	T	rs139167585	Modifier	Indel	0.167	0.089	0.131	0.004	0.197	0.14
*TTN*	Chr2	178795375	C	T	rs3754949	Modifier	Snv	0.042	0.206	0.059	0.117	0.013	0.065
*TTN*	Chr2	178759212	T	C	rs6705594	Modifier	Snv	0.000	0.004	-	0.001	-	0.002
*TTN*	Chr2	178629271	C	T	rs183058083	Modifier	Snv	0.083	0.003	-	-	-	-
*TTN*	Chr2	178675886	A	G	rs954235670	Modifier	Snv	0.000	-	-	-	-	-
*TTN*	Chr2	178769649	GTA	G	rs1445142745	Modifier	Indel	0.000	-	-	-	-	-
*TTN*	Chr2	178702075	T	TA	rs397517532	Modifier	Indel	0.009	0.222	0.159	0.028	0.038	0.186
*TTN*	Chr2	178749332	T	C	rs16866490	Modifier	Snv	0.000	0.02	0.003	-	-	-
*TTN*	Chr2	178752043	GAA	G	rs397517802	Modifier	Indel	0.480	0.031	0.01	0.001	0.003	0.004
*TTN*	Chr2	178538866	G	A	rs2303539	Modifier	Snv	0.042	0.002	0.143	0.451	0.139	0.235
*TTN*	Chr2	178786227	G	A	rs6715901	Modifier	Snv	0.414	0.148	0.383	0.133	0.512	0.273
*TTN*	Chr2	178751267	T	C	rs922986	Modifier	Snv	0.984	0.093	0.003	-	-	-
*TTN*	Chr2	178605320	C	A	rs2303833	Modifier	Snv	0.000	0.126	0.023	0.156	0.021	0.057
*TTN*	Chr2	178718671	T	C	rs62178978	Modifier	Snv	0.149	0.268	0.196	0.438	0.16	0.274
*TTN*	Chr2	178783096	C	T	rs60305852	Modifier	Snv	0.066	0.153	0.055	0.117	0.009	0.049
*TTN*	Chr2	178781004	C	A	rs12464703	Modifier	Snv	0.378	0.002	0.171	0.193	0.049	0.014
*TTN*	Chr2	178621439	TTAGA AATAAA	T	rs762765150	Modifier	Indel	0.031	-	-	-	-	-
*TTN*	Chr2	178746984	C	A	rs66677602	Modifier	Snv	0.438	0.002	0.17	0.199	0.048	0.017
*TTN*	Chr2	178647040	GTATA	GTA	rs1459735441	Modifier	Indel	0.000	-	-	-	-	-
*TTN*	Chr2	178677172	C	G	rs2742351	Modifier	Snv	0.096	0.221	0.343	0.235	0.255	0.113
*TTN*	Chr2	178749235	C	T	rs72648903	Modifier	Snv	0.000	0.07	0.003	-	-	-
*TTN*	Chr2	178640639	T	C	rs373511249	Modifier	Snv	0.000	0.002	-	-	-	-
*TTN*	Chr2	178802363	C	T	rs3816849	Modifier	Snv	0.977	0.274	0.313	0.143	0.438	0.328
*TTN*	Chr2	178751168	A	G	rs1226362110	Modifier	Snv	0.000	-	-	-	-	-
*TTN*	Chr2	178804517	G	GA	rs200033767	Modifier	Indel	0.000	0.002	-	0.001	0.002	0.016
*TTN*	Chr2	178745968	C	T	rs72648915	Modifier	Snv	0.000	0.14	0.01	-	-	-
*TTN*	Chr2	178766635	A	C	rs3816782	Modifier	Snv	0.519	0.161	0.245	0.195	0.077	0.073
*TTN*	Chr2	178717435	C	T	rs62178977	Modifier	Snv	0.08	0.194	0.184	0.436	0.157	0.246
*TTN*	Chr2	178695441	T	C	rs73038323	Modifier	Snv	0.000	0.135	0.014	-	0.001	-
*TTN*	Chr2	178730850	GT	G	rs35147775 rs72394294	Modifier	Indel	0.000	0.143	0.155	0.024	0.039	0.195
*TTN*	Chr2	178751204	C	G	rs922985	Modifier	Snv	0.984	0.093	0.003	-	-	-
*TTN*	Chr2	178675176	C	A	rs2472751	Modifier	Snv	0.543	0.571	0.405	0.578	0.234	0.517
*TTN*	Chr2	178745750	A	G	rs16866488	Modifier	Snv	0.000	0.037	0.003	-	-	-
*TTN*	Chr2	178527353	A	T	rs16866373	Modifier	Snv	0.014	0.067	0.148	0.45	0.139	0.267
*TTN*	Chr2	178715798	C	T	rs10183361	Modifier	Snv	0.017	0.165	0.153	0.026	0.036	0.176
*TTN*	Chr2	178647165	T	G	rs77497147	Modifier	Snv	0.014	0.001	0.036	0.028	-	0.001
*TTN*	Chr2	178701419	T	G	rs2251987	Modifier	Snv	1.00	0.131	0.0012	-	-	-
*TTN*	Chr2	178746953	G	A	rs72648911	Modifier	Snv	0.000	0.172	0.01	-	-	-
*TTN*	Chr2	178795303	T	C	rs3754950	Modifier	Snv	0.070	0.206	0.061	0.117	0.013	0.066
*TTN*	Chr2	178636905	C	T	rs570189553	Modifier	Snv	0.000	0.002	-	-	-	-
*TTN*	Chr2	178611738	G	A	rs67636125	Modifier	Snv	0.083	0.033	0.009	-	-	-
*TTN*	Chr2	178621452	A	T	rs760207565	Modifier	Snv	0.031	-	-	-	-	-
*TTN*	Chr2	178692111	C	T	rs372575606	Modifier	Snv	0.083	-	-	-	-	-
*TTN*	Chr2	178769664	TA	T	rs573000455	Modifier	Indel	0.050	0.008	0.001	0.006	-	-
*TTN*	Chr2	178615014	G	A	rs16866416	Modifier	Snv	0.185	0.1	0.151	0.023	0.033	0.181
*TTN*	Chr2	178683133	A	G	rs2303828	Modifier	Snv	0.125	0.265	0.202	0.459	0.176	0.304
*TTN*	Chr2	178767983	T	C	rs2291313	Modifier	Snv	0.086	0.408	0.483	0.332	0.776	0.555
*TTN*	Chr2	178577565	TA	T	rs148238009 rs796112072	Modifier	Indel	0.167	0.064	0.063	0.006	0.105	0.102
*TTN*	Chr2	178549906	C	A	rs890578	Modifier	Snv	0.014	0.278	0.199	0.63	0.171	0.314
*TTN*	Chr2	178649196	A	T	rs7606485	Modifier	Snv	0.397	0.098	0.151	0.023	0.033	0.181
*TTN*	Chr2	178769649	GTA	G	rs769907387	Modifier	Indel	0.000	-	-	-	-	-
*TTN*	Chr2	178540059	A	T	rs2288325	Modifier	Snv	0.095	0.148	0.157	0.454	0.138	0.269
*TTN*	Chr2	178621451	G	GT	rs750785162	Modifier	Indel	0.031	-	-	-	-	-
*TTN*	Chr2	178751160	T	C	rs922984	Modifier	Snv	0.135	0.425	0.359	0.225	0.086	0.218
*TTN*	Chr2	178733592	G	A	rs67039990	Modifier	Snv	0.083	0.054	0.01	-	-	-
*TTN*	Chr2	178602603	GA	G	rs778274900	Modifier	Indel	0.040	-	-	-	-	-
*PSCA*	Chr8	142681549	C	T	rs3736003	Modifier	Snv	0.027	0.179	0.020	0.115	0.014	0.041
*PSCA*	Chr8	142682339	T	G	rs1045547	Modifier	Snv	0.541	0.355	0.507	0.341	0.447	0.407
*PSCA*	Chr8	142682200	C	G	rs2976393	Modifier	Snv	0.813	0.355	0.509	0.341	0.448	0.407
*PSCA*	Chr8	142682583	G	A	rs2976396	Modifier	Snv	0.784	0.354	0.507	0.341	0.447	0.407
*PSCA*	Chr8	142682540	G	A	rs1045574	Modifier	Snv	0.789	0.354	0.507	0.342	0.447	0.407
*PSCA*	Chr8	142682204	C	T	rs2976394	Modifier	Snv	0.813	0.354	0.507	0.340	0.447	0.407
*PSCA*	Chr8	142682683	C	G	rs1045605	Modifier	Snv	0.700	0.354	0.507	0.341	0.447	0.408
*PSCA*	Chr8	142682272	G	A	rs10216533	Modifier	Snv	0.813	0.361	0.507	0.341	0.447	0.407
*PSCA*	Chr8	142681514	G	A	rs2976392	Modifier	Snv	0.265	0.368	0.507	0.341	0.446	0.407
*PSCA*	Chr8	142681306	C	A	rs2976391	Modifier	Snv	0.015	0.421	0.432	0.332	0.472	0.363
*PSCA*	Chr8	142680513	C	T	rs2294008	Modifier	Snv	0.813	0.368	0.504	0.342	0.447	0.407
*PSCA*	Chr8	142682332	G	A	rs2976395	Modifier	Snv	0.543	0.355	0.507	0.341	0.447	0.407
*CDH1*	Chr16	68737644	G	C	rs3743675	Modifier	Snv	0.500	0.363	0.223	0.23	0.119	0.207
*CDH1*	Chr16	68819472	G	A	rs35667437	Modifier	Snv	0.060	0.002	0.026	0.042	-	0.001
*CDH1*	Chr16	68823641	C	CA	rs1370519975	Modifier	Indel	0.000	0.002	0.072	0.07	0.044	0.064
*CDH1*	Chr16	68737515	C	CGC CCAC	rs147838237	Modifier	Indel	0.079	-	-	-	-	-
*TP53*	Chr17	7675954	A	G	rs926582621	Modifier	Snv	0.000	-	-	-	-	-
*TP53*	Chr17	7676325	#	C	rs1376609066	Modifier	Indel	1.00	0.482	0.39	0.318	0.378	0.472
*TP53*	Chr17	7676301	G	T	rs17883323	Modifier	Snv	0.156	0.116	0.053	0.073	0.058	0.069
*TP53*	Chr17	7676483	G	C	rs1642785	Modifier	Snv	0.563	0.508	0.307	0.418	0.288	0.495
*TP53*	Chr17	7674797	T	C	rs1625895	Modifier	Snv	0.000	0.315	0.091	0.024	0.143	0.189
*TP53*	Chr17	7675361	A	G	rs9895829	Modifier	Snv	0.016	0.115	0.053	0.075	0.059	0.084
*TP53*	Chr17	7675327	C	T	rs2909430	Modifier	Snv	1.00	0.286	0.088	0.024	0.142	0.185
*TP53*	Chr17	7673642	A	C	rs966675626	Modifier	Snv	0.000	-	-	-	-	-

(-) No annotation; (*) Minor allele frequencies; (#) CCCCCAGCCCTCCAGGT; INDG: Indigenous Amazonian population, AFR: African population, AMR: American population, EAS: East Asian population, EUR: European population, SAS: South Asian population.

**Table 2:** Description of new variants found in the Indigenous population from the Brazilian Amazon to genes relevant to increased susceptibility to gastric cancer.

Gene	Chromosome *	Position	Var Type	Region Detailed	Reference	Variant	Impact	Change Protein	Alteration
*TTN*	Chr2	178614388	Snv	Intron	A	C	Modifier	c.49049-40T>G	-
*TTN*	Chr2	178640679	Snv	Intron	A	G	Modifier	c.40634-49T>C	-
*TTN*	Chr2	178605617	Snv	Non-synonymous Coding	G	C	Moderate	p.Pro17893Arg	Missense
*TTN*	Chr2	178557925	Snv	Synonymous coding	G	A	Low	p.Gly29143Gly	Silent
*TTN*	Chr2	178662246	Snv	Intron	G	T	Modifier	c.37040-24C>A	-
*TTN*	Chr2	178738231	Snv	Non-synonymous Coding	T	C	Moderate	p.Tyr4741Cys	Missense
*TTN*	Chr2	178617063	Snv	Intron	G	C	Modifier	c.47876-50C>G	-
*TTN*	Chr2	178597932	Indel	Frame shift	CA	C	High	p.Asp19079fs	-
*TTN*	Chr2	178735467	Snv	Intron	C	T	Modifier	c.14935+44G>A	-
*TTN*	Chr2	178698916	Indel	Splice site region	TA	T	Low	c.30683-3delT	-
*TTN*	Chr2	178701523	Snv	Splice site region	C	A	Low	c.30598+5G>T	-
*TTN*	Chr2	178701499	Snv	Intron	A	G	Modifier	c.30598+29T>C	-
*TTN*	Chr2	178706876	Snv	Non-synonymous Coding	A	G	Moderate	p.Ile9707Thr	Missense
*TTN*	Chr2	178735471	Snv	Intron	G	T	Modifier	c.14935+40C>A	-
*TTN*	Chr2	178651537	Indel	Splice site acceptor	CTA	C	High	c.39464-3_39464-2delTA	-
*TTN*	Chr2	178718563	Snv	Non-synonymous Coding	A	T	Moderate	p.Ser8181Arg	Missense
*CDH1*	Chr16	68737646	Indel	Intron	T	TCC	Modifier	c.48+183_48+184insCC	-

(-) No annotation; (*) Reference genome to chromosomal location obtained from the GH38 of the human genome from the Human Genome Project.

## Data Availability

Not applicable.

## Supplementary Materials

The following supporting information can be downloaded at: https://www.mdpi.com/article/10.3390/jpm13091364/s1. Table S1. Descriptions of the variants in the *TTN*, *PSCA*, *PRKAA1*, *CDH1* and *TP53* genes according to the impact high, modifier and moderate, in addition to continental populations (African (AFR), American population (AMR), East Asian (EAS), European (EUR), and South Asian (SAS)) described in the 1000 genomes database. Table S2. Pairwise comparison (*p*-value) to significative results of allelic frequencies in Indigenous Amazonian population (INDG) and continental population (African (AFR), American population (AMR), East Asian (EAS), European (EUR), and South Asian (SAS)) described in the 1000 genomes database. Table S3. List of abreviations and acronyms.

## References

[1] Andrade R.B., Cohen-Paes A.d.N., Leal D.F.d.V.B., Pantoja K.B.C.C., Gellen L.P.A., de Carvalho D.C., de Souza T.P., Fernandes M.R., de Assumpcão P.P., Burbano R.M.R. (2023). Impact of pri-let-7a-1 rs10739971 for gastric cancer predisposition in an amazon region. Genes.

[2] Cancer National Institute. https://www.inca.gov.br/publicacoes/livros/estimativa-2023-incidencia-de-cancer-no-brasil.

[3] Karalis J.D., Ju M.R., Mansour J.C., Polanco P.M., Yopp A.C., Zeh H.J., Porembka M.R., Wang S.C. (2021). The presentation of Hispanic gastric cancer patients varies by location of patient ancestry. J. Surg Oncol..

[4] Yao Q., Qi X., Cheng W., Xie S.-H. (2019). A Comprehensive assessment of the racial and ethnic disparities in the incidence of gastric cancer in the United States, 1994–2014. Cancer Res. Treat..

[5] Rodrigues J.C.G., de Souza T.P., Pastana L.F., dos Santos A.M.R., Fernandes M.R., Pinto P., Wanderley A.V., de Souza S.J., Kroll J.E., Pereira A.L. (2020). Identification of NUDT15 gene variant in Amazonian Amerindians and admixed individuals from northern Brazil. PLoS ONE.

[6] Fernandes M.R., Rodrigues J.C.G., Dobbin E.A.F., Pastana L.F., da Costa D.F., Barra W.F., Modesto A.A.C., de Assumpção P.B., da Costa Silva A.L., Santos S.E.B.D. (2021). Influence of FPGS, ABCC4, SLC29A1 and MTHFR genes on the pharmacogenomics of fluorpyrimidines in patients with gastrointestinal cancer from Brazilian Amazon. Cancer Chemother. Pharmacol..

[7] Cardoso de Carvalho D., Pereira Colares Leitão L., Mello Junior F.A.R., Vieira Wanderley A., Souza T.P.d., Borges Andrade de Sá R., Cohen-Paes A., Rodrigues Fernandes M., Santos S., Salim Khayat A. (2020). Association between the TPMT*3C (rs1142345) polymorphism and the risk of death in the treatment of acute lymphoblastic leukemia in children from the brazilian amazon region. Genes.

[8] Cohen-Paes A.d.N., de Alcântara A.L., Moreira F.C., Fernandes M.R., Pantoja K.B.C.C., Carvalho D.C.d., Guerreiro J.F., Ribeiro-dos-Santos Â., Santos S.E.B.d., Assumpção P.P.D. (2022). Molecular epidemiology in amerindians of the brazilian amazon revels new genetic variants in DNA repais genes. Genes.

[9] Li Q., Guan R., Qiao Y., Liu C., He N., Zhang X., Jia X., Sun H., Yu J., Xu L. (2017). Association between the BRCA2 rs144848 polymorphism and cancer susceptibility: A meta-analysis. Oncotarget.

[10] Gu D., Zheng R., Xin J., Li S., Chu H., Gong W., Qiang F., Zhang Z., Wang M., Du M. (2018). Evaluation of GWAS-identified genetic variants for gastric cancer survival. EBio Med..

[11] Shenoy S. (2019). CDH1 (E-Cadherin) Mutation and gastric cancer: Genetics, molecular mechanisms and guidelines for management. Cancer Manag. Res..

[12] Hansford S. (2015). Hereditary Diffuse Gastric Cancer Syndrome: CDH1 Mutations and Beyond. JAMA Oncol..

[13] Sambrook J., Fritsch E.F., Maniatis T. (1989). Molecular Cloning: A Laboratory Manual.

[14] Cohen-Paes A.d.N., de Carvalho D.C., Pastana L.F., Dobbin E.A.F., Moreira F.C., de Souza T.P., Fernandes M.R., Leal D.F.d.V.B., de Sá R.B.A., de Alcântara A.L. (2022). Characterization of PCLO gene in Amazonian Native American populations. Genes.

[15] Moore S.P., Formam D., Piñeros M., Fernández S.M., de Oliveira Santos M., Bray F. (2014). Cancer in indigenous people in Latin American and the Caribbean: A review cancer. Cancer Med..

[16] Tishkoff S.A., Verrelli B.C. (2003). Patterns of human genetic diversity: Implications for human evolutionary history and disease. Annu. Rev. Genom. Hum. Genet..

[17] Zheng Q.-X., Wang J., Gu X.-Y., Huang C.-H., Chen C., Hong M., Chen Z. (2021). TTN-AS1 as a potential diagnostic and prognostic biomarker for multiple cancers. Biomed. Pharmacother..

[18] Hu X., Wang Z., Wang Q., Chen K., Han Q., Bai S., Du J., Chen W. (2021). Molecular classification reveals the diverse genetic and prognostic features of gastric cancer: A multi-omics consensus ensemble clustering. Biomed. Pharmacother..

[19] Dong Y., Song N., Wang J., Shi L., Zhang Z., Du J. (2022). Driver gene alterations in malignant progression of gastric cancer. Front. Oncol..

[20] Wang H., Shen L., Li Y., Lv J. (2020). Integrated characterization of cancer genes identifies key molecular biomarkers in stomach adenocarcinoma. J. Clin. Pathol..

[21] Floris M., Pira G., Castiglia P., Idda M.L., Steri M., De Miglio M.R., Muroni M.R. (2022). Impact on breast cancer susceptibility and clinicopathological traits of common genetic polymorphisms in TP53, MDM2 and ATM genes in Sardinian women. Oncol. Lett..

[22] Singh R.D., Patel K., Patel J.B., Patel P.S. (2023). Association of interactions between metabolic ‘caretaker’ genes, p53, MDM2, and tobacco use with the risk of oral cancer: A multifactor dimensionality reduction approach. Asian Pac. J. Cancer Prev..

[23] Donehower L.A., Soussi T., Korkut A., Liu Y., Schultz A., Cardenas M., Wheeler D.A. (2019). Integrated analysis of TP53 gene and pathway alterations in the cancer genome atlas. Cell Rep..

[24] Gargallo P., Yáñez Y., Segura V., Juan A., Torres B., Balaguer J., Cañete A. (2020). Li-Fraumeni syndrome heterogeneity. Clin. Transl. Oncol..

[25] Maxwell K.N., Cheng H.H., Powers J., Gulati R., Ledet E.M., Morrison C., Le A., Hausler R., Stopfer J., Hyman S. (2022). Inherited TP53 variants and risk of prostate cancer. Eur. Urol..

[26] Gaspar P.A., Hutz M.H., Salzano F.M., Weimer T.A. (2001). TP53 polymorphisms and haplotypes in South Amerindians and neo-Brazilians. Ann. Hum. Biol..

[27] Luo W., Fedda F., Lynch P., Tan D. (2018). CDH1 Gene and hereditary diffuse gastric cancer syndrome: Molecular and histological alterations and implications for diagnosis and treatment. Front. Pharmacol..

