# Supplementary material for: Molecular Profile of Variants in CDH1, TP53, PSCA, PRKAA1, and TTN Genes Related to Gastric Cancer Susceptibility in Amazonian Indigenous Populations
Source: J Pers Med. 2023 Sep 8;13(9):1364. doi: 10.3390/jpm13091364 (PMC10532670; doi:10.3390/jpm13091364)
Supplement: Supplementary file 1 [file jpm-13-01364-s001.zip › jpm-2550721-supplementary.pdf]

Table S1. Descriptions of the variants in the *TTN*, *PSCA*, *PRKAA1*, *CDH1* and *TP53* genes according to the impact high, modifier and moderate, in addition to continental populations (African (AFR), American population (AMR), East Asian (EAS), European (EUR), and South Asian (SAS)) described in the 1000 genomes database.

Gene	Chromosome	Position	Var Type	Reference	Variant	SNP Id	Impact	INDG*	AFR*	AMR*	EAS*	EUR*	SAS*
TTN	Chr2	178597932	Indel	CTA	C	.	High	0.000	-	-	-	-	-
TTN	Chr2	178704146	Snv	C	T	rs556408709	High	0.000	-	0.001	-	-	-
TTN	Chr2	178651537	Snv	C	C	.	High	0.000	-	-	-	-	-
TTN	Chr2	178634311	Snv	A	G	rs7590037	Modifier	0.392	0.157	0.156	0.024	0.033	0.180
TTN	Chr2	178745892	Snv	C	G	rs16866489	Modifier	0.019	0.020	0.003	-	-	-
TTN	Chr2	178614388	Snv	A	C	.	Modifier	0.000	-	-	-	-	-
TTN	Chr2	178685342	Snv	T	C	rs16866434	Modifier	0.000	0.033	0.017	0.012	0.013	0.017
TTN	Chr2	178722960	Snv	T	C	rs2742327	Modifier	0.568	0.612	0.399	0.659	0.225	0.474
TTN	Chr2	178536584	Snv	C	CA	rs397517782	Modifier	0.100	0.004	0.006	-	0.008	0.005
TTN	Chr2	178717878	Snv	A	G	rs6750145	Modifier	0.028	0.298	0.196	0.436	0.160	0.274
TTN	Chr2	178669342	Snv	G	A	rs181914612	Modifier	0.000	0.003	0.003	-	-	-
TTN	Chr2	178730861	Snv	TA	T	rs376726021	Modifier	0.083	-	0.026	0.002	0.019	0.004
TTN	Chr2	178640679	Snv	A	G	.	Modifier	0.000	-	-	-	-	-
TTN	Chr2	178678713	Snv	A	C	rs184108456	Modifier	0.000	-	0.012	-	-	-
TTN	Chr2	178783080	Indel	AAAT	A	rs752207098	Modifier	0.000	-	-	-	-	-
TTN	Chr2	178748420	Snv	G	C	rs75785339	Modifier	0.017	0.141	0.010	-	-	-
TTN	Chr2	178592719	Snv	A	G	rs72646841	Modifier	0.000	0.135	0.050	0.180	0.045	0.063
TTN	Chr2	178546938	Snv	A	G	rs2288327	Modifier	0.014	0.289	0.199	0.630	0.172	0.319
TTN	Chr2	178730850	Indel	GT	GTT	rs35147775<br>rs72394294	Modifier	0.000	-	-	-	-	-
TTN	Chr2	178647040	Indel	GTA	G	rs10580462<br>rs1401721480	Modifier	0.000	-	-	-	-	-
TTN	Chr2	178730850	Indel	GT	G	rs35147775<br>rs72394294	Modifier	0.000	-	-	-	-	-
TTN	Chr2	178730390	Snv	C	G	rs17076	Modifier	0.065	0.286	0.339	0.669	0.189	0.277
TTN	Chr2	178746176	Snv	A	G	rs529571649	Modifier	0.000	-	-	-	0.001	-
TTN	Chr2	178681069	Snv	A	G	rs72650039	Modifier	0.083	0.003	0.003	-	-	-
TTN	Chr2	178766637	Indel	AC	A	rs140501763	Modifier	0.426	-	-	-	-	-
TTN	Chr2	178702075	Indel	T	TA	rs397517532	Modifier	0.000	0.222	0.159	0.028	0.038	0.186

TTN	Chr2	178728858	Snv	T	C	rs16866476	Modifier	0.000	0.020	0.003	-	-	-
TTN	Chr2	178748531	Snv	A	C	rs72648906	Modifier	0.000	0.076	0.004	-	-	-
TTN	Chr2	178605617	Snv	G	C	.	Modifier	0.333	-	-	-	-	-
TTN	Chr2	178783097	Snv	G	A	rs72647864	Modifier	0.025	-	0.053	-	0.032	0.022
TTN	Chr2	178689003	Indel	ATTTTTT	A	rs1242467782	Modifier	0.250	0.284	0.029	-	0.038	0.122
TTN	Chr2	178653350	Snv	A	G	rs13420457	Modifier	0.083	-	-	-	-	-
TTN	Chr2	178779207	Snv	A	G	rs2291301	Modifier	0.930	0.151	0.050	0.117	0.008	0.041
TTN	Chr2	178689190	Snv	G	A	rs2627038	Modifier	0.419	0.509	0.231	0.129	0.076	0.277
TTN	Chr2	178688666	Snv	C	T	rs369265969	Modifier	0.000	-	-	-	-	-
TTN	Chr2	178752043	Indel	GAA	G	rs397517802	Modifier	0.000	-	-	-	-	-
TTN	Chr2	178769662	Indel	TA	T	rs570467105	Modifier	0.051	0.008	0.001	0.006	-	-
TTN	Chr2	178804500	Indel	CTGGAG	C	rs3830329	Modifier	0.106	0.090	0.045	0.117	0.009	0.039
TTN	Chr2	178647040	Indel	GTATA	G	rs1459735441 rs786205393	Modifier	0.000	-	-	-	-	-
TTN	Chr2	178535858	Indel	GA	G	rs749872538	Modifier	0.146	0.003	0.002	0.001	0.001	0.002
TTN	Chr2	178789586	Snv	A	G	rs13421990	Modifier	0.179	0.012	0.150	0.016	0.035	0.162
TTN	Chr2	178779159	Snv	A	G	rs2291302	Modifier	0.930	0.151	0.050	0.117	0.008	0.041
TTN	Chr2	178706970	Snv	C	T	rs10203085	Modifier	0.016	0.102	0.147	0.026	0.036	0.177
TTN	Chr2	178750225	Snv	G	A	rs10803917	Modifier	0.984	0,093	0,003	-	-	-
TTN	Chr2	178654184	Indel	TAGC	T	rs139167585 rs372316744	Modifier	0.167	0.089	0.131	0.004	0.197	0.14
TTN	Chr2	178795375	Snv	C	T	rs3754949	Modifier	0.042	0.206	0.059	0.117	0.013	0.065
TTN	Chr2	178759212	Snv	C	T	rs6705594	Modifier	0.000	0.004	-	0.001	-	0.002
TTN	Chr2	178629271	Snv	C	T	rs183058083	Modifier	0.083	0.003	-	-	-	-
TTN	Chr2	178675886	Snv	A	G	rs954235670	Modifier	0.000	-	-	-	-	-
TTN	Chr2	178735467	Snv	C	T	.	Modifier	0.000	-	-	-	-	-
TTN	Chr2	178769649	Indel	GTA	G	rs1445142745 rs794729235	Modifier	0.000	-	-	-	-	-
TTN	Chr2	178617063	Snv	G	C	.	Modifier	0.083	-	-	-	-	-
TTN	Chr2	178702075	Snv	T	TA	rs397517532	Modifier	0.009	0.222	0.159	0.028	0.038	0.186
TTN	Chr2	178749332	Snv	T	C	rs16866490	Modifier	0.000	0.02	0.003	-	-	-
TTN	Chr2	178752043	Indel	GAA	GA	rs397517802	Modifier	0.480	0.031	0.01	0.001	0.003	0.004
TTN	Chr2	178538866	Snv	G	A	rs2303539	Modifier	0.042	0.002	0.143	0.451	0.139	0.235
TTN	Chr2	178786227	Snv	G	A	rs6715901	Modifier	0.414	0.148	0.383	0.133	0.512	0.273

TTN	Chr2	178751267	Snv	C	T	rs922986	Modifier	0.984	0.093	0.003	-	-	-
TTN	Chr2	178605320	Snv	C	A	rs2303833	Modifier	0.000	0.126	0.023	0.156	0.021	0.057
TTN	Chr2	178718671	Snv	T	C	rs62178978	Modifier	0.149	0.268	0.196	0.438	0.16	0.274
TTN	Chr2	178783096	Snv	C	T	rs60305852	Modifier	0.066	0.153	0.055	0.117	0.009	0.049
TTN	Chr2	178781004	Snv	C	A	rs12464703	Modifier	0.378	0.002	0.171	0.193	0.049	0.014
TTN	Chr2	178621439	Indel	TTAGAA ATAAA	T	rs762765150	Modifier	0.031	-	-	-	-	-
TTN	Chr2	178746984	Snv	C	A	rs66677602	Modifier	0.438	0.002	0.17	0.199	0.048	0.017
TTN	Chr2	178647040	Indel	GTATA	GTA	rs1459735441 rs786205393	Modifier	0.000	-	-	-	-	-
TTN	Chr2	178677172	Snv	G	C	rs2742351	Modifier	0.096	0.221	0.343	0.235	0.255	0.113
TTN	Chr2	178749235	Snv	C	T	rs72648903	Modifier	0.000	0.07	0.003	-	-	-
TTN	Chr2	178640639	Snv	T	C	rs373511249	Modifier	0.000	0.002	-	-	-	-
TTN	Chr2	178802363	Snv	T	C	rs3816849	Modifier	0.977	0.274	0.313	0.143	0.438	0.328
TTN	Chr2	178751168	Snv	A	G	rs1226362110	Modifier	0.000	-	-	-	-	-
TTN	Chr2	178804517	Indel	G	GA	rs200033767	Modifier	0.000	0.002	-	0.001	0.002	0.016
TTN	Chr2	178745968	Snv	C	T	rs72648915	Modifier	0.000	0.14	0.01	-	-	-
TTN	Chr2	178766635	Snv	A	C	rs3816782	Modifier	0.519	0.161	0.245	0.195	0.077	0.073
TTN	Chr2	178717435	Snv	C	T	rs62178977	Modifier	0.08	0.194	0.184	0.436	0.157	0.246
TTN	Chr2	178695441	Snv	T	C	rs73038323	Modifier	0.000	0.135	0.014	-	0.001	-
TTN	Chr2	178751204	Snv	C	G	rs922985	Modifier	0.984	0.093	0.003	-	-	-
TTN	Chr2	178675176	Snv	C	A	rs2472751	Modifier	0.543	0.571	0.405	0.578	0.234	0.517
TTN	Chr2	178745750	Snv	A	G	rs16866488	Modifier	0.000	0.037	0.003	-	-	-
TTN	Chr2	178527353	Snv	A	T	rs16866373	Modifier	0.014	0.067	0.148	0.45	0.139	0.267
TTN	Chr2	178715798	Snv	C	T	rs10183361	Modifier	0.017	0.165	0.153	0.026	0.036	0.176
TTN	Chr2	178647165	Snv	T	G	rs77497147	Modifier	0.014	0.001	0.036	0.028	-	0.001
TTN	Chr2	178701419	Snv	G	T	rs2251987	Modifier	1.00	0.131	0.0012	-	-	-
TTN	Chr2	178746953	Snv	G	A	rs72648911	Modifier	0.000	0.172	0.01	-	-	-
TTN	Chr2	178795303	Snv	T	C	rs3754950	Modifier	0.070	0.206	0.061	0.117	0.013	0.066
TTN	Chr2	178636905	Snv	C	T	rs570189553	Modifier	0.000	0.002	-	-	-	-
TTN	Chr2	178611738	Snv	G	A	rs67636125	Modifier	0.083	0.033	0.009	-	-	-
TTN	Chr2	178701499	Snv	A	G	.	Modifier	0.000	-	-	-	-	-
TTN	Chr2	178621452	Snv	A	T	rs760207565	Modifier	0.031	-	-	-	-	-
TTN	Chr2	178692111	Snv	C	T	rs372575606	Modifier	0.083	-	-	-	-	-

TTN	Chr2	178769664	Indel	TA	T	rs573000455	Modifier	0.050	0.008	0.001	0.006	-	-
TTN	Chr2	178615014	Snv	G	A	rs16866416	Modifier	0.185	0.1	0.151	0.023	0.033	0.181
TTN	Chr2	178577565	Indel	GT	G	rs35147775<br>rs72394294	Modifier	0.000	0.143	0.155	0.024	0.039	0.195
TTN	Chr2	178683133	Snv	A	G	rs2303828	Modifier	0.125	0.265	0.202	0.459	0.176	0.304
TTN	Chr2	178767983	Snv	T	C	rs2291313	Modifier	0.086	0.408	0.483	0.332	0.776	0.555
TTN	Chr2	178577565	Indel	TA	T	rs148238009<br>rs796112072	Modifier	0.167	0.064	0.063	0.006	0.105	0.102
TTN	Chr2	178735471	Snv	G	T	.	Modifier	0.000	-	-	-	-	-
TTN	Chr2	178549906	Snv	C	A	rs890578	Modifier	0.014	0.278	0.199	0.63	0.171	0.314
TTN	Chr2	178649196	Snv	A	T	rs7606485	Modifier	0.397	0.098	0.151	0.023	0.033	0.181
TTN	Chr2	178769649	Indel	GTATA	G	rs769907387	Modifier	0.000	-	-	-	-	-
TTN	Chr2	178540059	Snv	A	T	rs2288325	Modifier	0.095	0.148	0.157	0.454	0.138	0.269
TTN	Chr2	178621451	Indel	G	GT	rs750785162	Modifier	0.031	-	-	-	-	-
TTN	Chr2	178751160	Snv	C	T	rs922984	Modifier	0.135	0.425	0.359	0.225	0.086	0.218
TTN	Chr2	178733592	Snv	G	A	rs67039990	Modifier	0.083	0.054	0.01	-	-	-
TTN	Chr2	178602603	Indel	GA	G	rs778274900	Modifier	0.040	-	-	-	-	-
TTN	Chr2	178533782	Snv	C	A	rs3731752	Moderate	0.0431	0.085	0.04	0.172	0.032	0.047
TTN	Chr2	178778905	Snv	T	C	rs16866531	Moderate	0.0833	0.142	0.012	-	-	-
TTN	Chr2	178599800	Snv	T	C	rs1001238	Moderate	0.2556	0.431	0.592	0.275	0.747	0.441
TTN	Chr2	178549663	Snv	C	T	rs375190050	Moderate	0	-	-	-	-	-
TTN	Chr2	178795185	Snv	G	A	rs16866538	Moderate	0.0476	0.197	0.098	0.588	0.048	0.217
TTN	Chr2	178693639	Snv	T	C	rs2042995	Moderate	0.5156	0.588	0.419	0.568	0.246	0.516
TTN	Chr2	178739433	Snv	T	G	rs1883085	Moderate	0.4375	0.003	0.17	0.193	0.048	0.017
TTN	Chr2	178607551	Snv	G	C	rs1008171003	Moderate	0.0086	-	-	-	-	-
TTN	Chr2	178739639	Snv	T	G	rs2562829	Moderate		0.002	0.17	0.193	0.048	0.017
TTN	Chr2	178759031	Snv	C	T	rs2291310	Moderate	0.1406	0.161	0.33	0.215	0.085	0.217
TTN	Chr2	178653276	Snv	A	G	rs2562847	Moderate	0.1667	0.042	0.182	0.125	0.064	0.22
TTN	Chr2	178689578	Snv	C	T	rs2244492	Moderate	0.4453	0.607	0.35	0.168	0.383	0.482
TTN	Chr2	178631063	Snv	T	G	rs796538475	Moderate	0	-	-	-	-	-
TTN	Chr2	178571293	Snv	G	A	rs744426	Moderate	0.0703	0.067	0.146	0.45	0.137	0.267
TTN	Chr2	178720453	Snv	C	T	rs748511026	Moderate	0	-	-	-	-	-
TTN	Chr2	178541464	Snv	C	T	rs3731749	Moderate	0.0703	0.067	0.148	0.451	0.138	0.269
TTN	Chr2	178706721	Snv	A	G	rs4893852	Moderate	0.431	0.223	0.156	0.026	0.036	0.177

TTN	Chr2	178528384	Snv	A	G	rs16866378	Moderate	0	0.135	0.04	0.174	0.032	0.048
TTN	Chr2	178564110	Snv	C	T	rs555414240	Moderate	0.0086	-	-	0.001	-	-
TTN	Chr2	178724514	Snv	G	A	rs17355446	Moderate	0.0714	0.002	0.144	0.002	0.03	0.126
TTN	Chr2	178576952	Snv	G	T	rs72646882	Moderate	0	0.005	-	-	-	-
TTN	Chr2	178717810	Snv	G	T	rs2627043	Moderate	0.5745	0.464	0.379	0.658	0.225	0.473
TTN	Chr2	178530833	Snv	G	A	rs16866380	Moderate	0.0431	0.089	0.04	0.172	0.032	0.048
TTN	Chr2	178569789	Snv	C	T	rs3813243	Moderate	0	0.085	0.042	0.174	0.033	0.048
TTN	Chr2	178633315	Snv	T	C	rs6723526	Moderate	0.0833	0.006	0.062	0.005	0.1	0.1
TTN	Chr2	178563334	Snv	C	T	rs11896637	Moderate	0	0.126	0.016	-	0.001	-
TTN	Chr2	178612313	Snv	C	T	rs148018042	Moderate	0	-	-	-	0.001	0.001
TTN	Chr2	178551932	Snv	C	T	rs11887722	Moderate	0.0089	0.127	0.016	-	0.001	-
TTN	Chr2	178539901	Snv	T	A	rs72648270	Moderate	0	0.002	0.029	0.149	0.03	0.011
TTN	Chr2	178741811	Snv	G	A	rs2627037	Moderate	0.8672	0.453	0.36	0.213	0.087	0.221
TTN	Chr2	178549591	Snv	C	T	rs747122	Moderate	0.4237	0.273	0.179	0.025	0.049	0.197
TTN	Chr2	178696120	Snv	C	T	rs73038324	Moderate	0	0.138	0.016	-	0.001	-
TTN	Chr2	178580041	Snv	C	G	rs4145333	Moderate	1	0.019	0.003	-	-	-
TTN	Chr2	178710784	Snv	C	T	rs72648998	Moderate	0.0714	-	0.081	-	0.055	0.027
TTN	Chr2	178735921	Snv	C	T	rs2742347	Moderate	0.4531	0.228	0.192	0.193	0.052	0.044
TTN	Chr2	178621869	Snv	G	A	rs72677221	Moderate	0	-	0.012	0.001	-	-
TTN	Chr2	178650214	Indel	#	T	rs139512154	Moderate	0	-	-	-	-	-
TTN	Chr2	178575570	Snv	G	C	rs775589244	Moderate	0	-	-	-	-	-
TTN	Chr2	178717600	Snv	C	T	rs13390491	Moderate	0.129	0.194	0.183	0.436	0.157	0.246
TTN	Chr2	178764191	Snv	C	T	rs34819099	Moderate	0.0833	-	0.003	0.001	0.013	0.003
TTN	Chr2	178733443	Snv	C	T	rs66839174	Moderate	0.0833	0.011	0.004	-	-	-
TTN	Chr2	178720935	Snv	G	T	rs201003628	Moderate	0	-	-	-	-	-
TTN	Chr2	178777856	Snv	A	G	rs142317580	Moderate	0.0156	-	0.027	-	-	-
TTN	Chr2	178583666	Snv	G	A	rs55948748	Moderate	0	0.051	0.004	-	0.001	-
TTN	Chr2	178785681	Snv	G	A	rs35813871	Moderate	0.0833	0.086	0.15	0.001	0.216	0.069
TTN	Chr2	178713977	Snv	G	A	rs13398235	Moderate	0.4141	0.165	0.153	0.026	0.036	0.176
TTN	Chr2	178735910	Snv	C	T	rs752150323	Moderate	0.0833	-	-	-	-	-
TTN	Chr2	178609941	Snv	G	A	rs16866412	Moderate	0	0.087	0.022	0.156	0.021	0.057
TTN	Chr2	178539023	Snv	C	T	rs4894028	Moderate	0.4167	0.011	0.148	0.025	0.048	0.198
TTN	Chr2	178593967	Snv	C	T	rs397517636	Moderate	0	-	-	-	-	0.031
TTN	Chr2	178652497	Snv	C	A	rs768849222	Moderate	0.125	-	-	-	-	-

TTN	Chr2	178593864	Snv	C	T	rs2288569	Moderate	0.0776	0.067	0.151	0.454	0.14	0.278
TTN	Chr2	178536288	Snv	G	A	rs72629779	Moderate	0	0.05	0.004	-	-	-
TTN	Chr2	178566270	Snv	G	A	rs3731746	Moderate	0.1094	0.344	0.206	0.629	0.171	0.317
TTN	Chr2	178731454	Snv	G	C	rs16866477	Moderate	0	0.02	0.003	-	-	-
TTN	Chr2	178776633	Snv	G	A	rs75686037	Moderate	0.0469	0.001	0.024	0.017	-	-
TTN	Chr2	178537015	Snv	C	T	rs55742743	Moderate	0.0833	-	0.004	-	0.018	0.003
TTN	Chr2	178632291	Snv	G	A	rs12471771	Moderate	0.3828	-	0.117	-	-	-
TTN	Chr2	178779308	Snv	G	A	rs1552280	Moderate	0.9297	0.151	0.05	0.117	0.008	0.041
TTN	Chr2	178770209	Snv	C	T	rs2306636	Moderate	0.4375	0.002	0.173	0.19	0.051	0.042
TTN	Chr2	178547725	Snv	C	T	rs67665715	Moderate	0.0086	0.127	0.0016	-	0.001	-
TTN	Chr2	178586693	Snv	A	G	rs2042996	Moderate	0.5234	0.442	0.592	0.279	0.751	0.444
TTN	Chr2	178756224	Snv	T	C	rs7585334	Moderate	0.45	0.17	0.33	0.224	0.085	0.218
TTN	Chr2	178592420	Snv	G	A	rs16866406	Moderate	0.0703	0.067	0.153	0.457	0.139	0.278
TTN	Chr2	178571754	Snv	G	A	rs766741890	Moderate	0	-	-	-	-	-
TTN	Chr2	178531435	Snv	C	G	rs56308529	Moderate	0.0625	-	0.079	-	0.047	0.013
TTN	Chr2	178580212	Snv	C	T	rs2303838	Moderate	0.1186	0.343	0.215	0.631	0.185	0.342
TTN	Chr2	178718126	Snv	T	C	rs72648982	Moderate	0.0833	0.002	0.013	0.001	0.028	0.02
TTN	Chr2	178764734	Snv	T	C	rs2291311	Moderate	0.1452	0.175	0.333	0.215	0.085	0.217
TTN	Chr2	178543986	Snv	A	G	rs62621236	Moderate	0.4063	0.146	0.164	0.026	0.052	0.199
TTN	Chr2	178722403	Snv	C	G	rs12693166	Moderate	0.125	0.202	0.184	0.438	0.158	0.247
TTN	Chr2	178536168	Snv	C	T	rs2278196	Moderate	0.0431	0.085	0.036	0.149	0.03	0.011
TTN	Chr2	178549435	Snv	T	C	rs16866391	Moderate	0.087	0.001	0.161	0.057	-	0.004
TTN	Chr2	178777248	Snv	C	T	rs12476289	Moderate	0.4375	0.002	0.171	0.189	0.049	0.015
TTN	Chr2	178714366	Snv	T	C	rs12693164	Moderate	0.125	0.194	0.186	0.437	0.156	0.261
TTN	Chr2	178712901	Snv	C	T	rs766095051	Moderate	0	-	-	-	-	-
TTN	Chr2	178574139	Snv	C	T	rs10164753	Moderate	0.4063	0.088	0.154	0.026	0.047	0.198
TTN	Chr2	178577190	Snv	T	C	rs72646881	Moderate	0	0.128	0.017	-	0.001	-
TTN	Chr2	178563160	Snv	C	T	rs150150605	Moderate	0.0086	0.001	-	-	-	-
TTN	Chr2	178773134	Snv	C	G	rs56142888	Moderate	0.3917	0.002	0.141	0.19	0.049	0.015
TTN	Chr2	178756626	Snv	G	A	rs57389274	Moderate	0	0.073	0.003	-	-	-
TTN	Chr2	178718769	Snv	T	G	rs16866465	Moderate	0.125	0.173	0.183	0.438	0.157	0.246
TTN	Chr2	178756622	Snv	T	G	rs79466278	Moderate	0.0833	0.011	0.04	-	-	-
TTN	Chr2	178556967	Snv	G	A	rs9808377	Moderate	0.5234	0.433	0.595	0.285	0.75	0.45
TTN	Chr2	178663311	Snv	A	C	rs12994774	Moderate	0.0833	-	-	-	-	-

TTN	Chr2	178663651	Snv	C	T	rs2163008	Moderate	0.4426	0.503	0.228	0.134	0.076	0.27
TTN	Chr2	178688168	Snv	C	T	rs72650028	Moderate	0	0.048	0.004	-	-	-
TTN	Chr2	178642294	Snv	G	A	rs769313634	Moderate	0	-	-	-	-	-
TTN	Chr2	178532834	Snv	C	T	rs3829747	Moderate	0.0703	0.067	0.148	0.448	0.139	0.268
TTN	Chr2	178562809	Snv	T	C	rs3829746	Moderate	0.5234	0.434	0.592	0.285	0.747	0.499
TTN	Chr2	178780128	Snv	T	C	rs10497520	Moderate	0.0484	0.592	0.491	0.783	0.143	0.456
TTN	Chr2	178550106	Snv	C	T	rs727504672	Moderate	0.0086	-	-	-	-	-
TTN	Chr2	178728625	Snv	C	T	rs11888217	Moderate	0.3984	0.087	0.151	0.024	0.036	0.191
TTN	Chr2	178773994	Snv	C	T	rs4894048	Moderate	0.3906	0.092	0.16	0.015	0.036	0.158
TTN	Chr2	178735681	Snv	C	T	rs184740744	Moderate	0.0156	-	0.027	-	-	0.001
TTN	Chr2	178664080	Snv	T	A	rs73973133	Moderate	0.0234	0.027	0.004	-	0.001	-
TTN	Chr2	178767769	Snv	T	C	rs4893853	Moderate	0.4063	0.075	0.154	0.027	0.035	0.173
TTN	Chr2	178609259	Snv	C	T	rs765525785	Moderate	0.0143	-	-	-	-	-
TTN	Chr2	178731053	Snv	A	G	rs532976769	Moderate	0	-	-	-	-	-
TTN	Chr2	178735836	Snv	T	C	rs2742348	Low	1.00	0.066	0.006	-	-	-
TTN	Chr2	178652522	Snv	G	A	rs1230759204	Low	0.000	-	-	-	-	-
TTN	Chr2	178575302	Snv	T	C	rs12464787	Low	0.083	0.078	0.117	0.042	0.305	0.206
TTN	Chr2	178713143	Snv	G	A	rs61232800	Low	0.018	0.089	0.006	-	-	-
TTN	Chr2	178531627	Snv	G	A	rs3829748	Low	0.070	0.148	0.157	0.450	0.138	0.268
TTN	Chr2	178773511	Snv	G	A	rs2291306	Low	0.167	0.081	0.071	0.022	0.095	0.106
TTN	Chr2	178557925	Snv	G	A	.	Low	0.000	-	-	-	-	-
TTN	Chr2	178534096	Snv	A	G	rs2857265	Low	0.070	0.148	0.157	0.451	0.139	0.268
TTN	Chr2	178722819	Snv	A	G	rs16866473	Low	0.398	0.089	0.153	0.026	0.036	0.190
TTN	Chr2	178720666	Snv	A	G	rs2562830	Low	0.070	0.513	0.392	0.660	0.225	0.473
TTN	Chr2	178570940	Snv	C	G	rs370480927	Low	0.009	0.002	-	-	-	-
TTN	Chr2	178530340	Snv	A	G	rs56207956	Low	0.000	-	-	-	-	-
TTN	Chr2	178607870	Snv	T	G	rs73036398	Low	0.009	0.077	0.016	-	0.002	-
TTN	Chr2	178570610	Snv	C	T	rs6732060	Low	0.414	0.241	0.180	0.026	0.053	0.199
TTN	Chr2	178733672	Snv	C	T	rs72648932	Low	0.018	0.088	0.006	-	-	-
TTN	Chr2	178556882	Snv	C	C	rs9808036	Low	0.405	0.038	0.153	0.023	0.048	0.197
TTN	Chr2	178614518	Snv	A	T	rs72677244	Low	0.018	0.085	0.023	0.157	0.027	0.057
TTN	Chr2	178724522	Snv	T	C	rs746178307	Low	0.000	-	-	-	-	-
TTN	Chr2	178766487	Snv	T	A	rs2291312	Low	0.836	0.077	0.327	0.214	0.084	0.215
TTN	Chr2	178729863	Snv	T	C	rs66523653	Low	0.083	0.054	0.010	-	-	-

TTN	Chr2	178779433	Indel	TA	T	rs2291308	Low	0.472	0.002	0.171	0.192	0.049	0.014
TTN	Chr2	178698916	Snv	C	T	rs368277751	Low	0.000	-	-	-	-	-
TTN	Chr2	178740453	Snv	A	T	rs746578	Low	0.073	0.185	0.159	0.470	0.138	0.234
TTN	Chr2	178567070	Snv	T	C	rs3731744	Low	0.044	0.133	0.042	0.177	0.033	0.049
TTN	Chr2	178764636	Snv	A	C	rs4894043	Low	0.984	0.092	0.006	-	-	-
TTN	Chr2	178541276	Snv	A	G	rs3731750	Low	0.318	0.439	0.598	0.287	0.751	0.450
TTN	Chr2	178562459	Snv	A	G	rs2366751	Low	0.523	0.433	0.592	0.285	0.747	0.449
TTN	Chr2	178782819	Snv	A	G	rs55863869	Low	0.000	0.002	0.171	0.194	0.056	0.015
TTN	Chr2	178612990	Snv	A	G	rs2115558	Low	0.017	0.089	0.023	0.158	0.021	0.057
TTN	Chr2	178740978	Snv	T	T	rs2742357	Low	0.438	0.002	0.17	0.193	0.048	0.017
TTN	Chr2	178698916	Snv	A	G	.	Low	0.000	-	-	-	-	-
TTN	Chr2	178777698	Snv	G	A	rs719201	Low	0.930	0.182	0.053	0.117	0.008	0.041
TTN	Chr2	178593275	Snv	A	G	rs2303836	Low	0.430	0.001	0.157	0.057	-	0.005
TTN	Chr2	178722052	Snv	C	A	rs16866469	Low	0.000	0.075	0.003	-	-	-
TTN	Chr2	178740453	Snv	C	A	rs746578	Low	0.256	0.002	0.170	0.193	0.048	0.017
TTN	Chr2	178740453	Snv	A	G	rs746578	Low	0.031	0.002	0.17	0.193	0.048	0.017
TTN	Chr2	178563059	Snv	G	A	rs569803719	Low	0.000	-	0.003	-	-	-
TTN	Chr2	178548383	Snv	C	A	rs3731748	Low	0.070	0.148	0.157	0.451	0.138	0.28
TTN	Chr2	178735467	Snv	G	A	.	Low	0.000	-	-	-	-	-
TTN	Chr2	178718590	Snv	G	A	rs72648978	Low	0.167	0.08	0.069	0.02	0.094	0.101
TTN	Chr2	178565810	Snv	G	A	rs55892928	Low	0.000	-	-	-	-	-
TTN	Chr2	178729135	Snv	G	T	rs72648950	Low	0.417	0.001	0.147	-	0.008	0.01
TTN	Chr2	178566443	Snv	T	C	rs10185798	Low	0.083	0.033	0.003	-	-	-
TTN	Chr2	178714485	Snv	G	A	rs2562838	Low	0.547	0.602	0.393	0.659	0.224	0.473
TTN	Chr2	178714003	Snv	G	A	rs2562839	Low	0.539	0.464	0.379	0.657	0.224	0.473
TTN	Chr2	178740015	Snv	C	T	rs1883084	Low	0.438	0.006	0.17	0.192	0.048	0.017
TTN	Chr2	178583121	Snv	T	G	rs4894029	Low	0.523	0.125	0.592	0.28	0.745	0.441
TTN	Chr2	178652498	Snv	T	A	rs776542838	Low	0.125	-	-	-	-	-
TTN	Chr2	178715095	Snv	T	C	rs2562836	Low	0.539	0.464	0.379	0.658	0.224	0.471
TTN	Chr2	178720539	Snv	C	T	rs2562831	Low	1.00	0.065	0.006	-	-	-
TTN	Chr2	178785974	Snv	A	G	rs6715406	Low	0.023	0.144	0.192	0.068	0.414	0.221
TTN	Chr2	178578813	Snv	T	C	rs10497517	Low	0.103	0.216	0.174	0.026	0.053	0.199
TTN	Chr2	178633576	Snv	T	C	rs16866425	Low	0.406	0.157	0.156	0.023	0.033	0.181
TTN	Chr2	178590480	Snv	T	C	rs2163009	Low	0.523	0.431	0.592	0.277	0.746	0.442

TTN	Chr2	178561679	Snv	G	A	rs73036373	Low	0.011	0.042	0.004	-	0.001	-
TTN	Chr2	178621192	Snv	A	C	rs61004744	Low	0.023	0.082	0.003	-	-	-
TTN	Chr2	178551829	Snv	G	A	rs11897366	Low	0.414	0.274	0.183	0.026	0.053	0.199
TTN	Chr2	178531033	Snv	G	A	rs3829749	Low	0.000	-	-	-	-	-
TTN	Chr2	178589667	Snv	C	T	rs1560221	Low	0.531	0.432	0.591	0.277	0.746	0.442
TTN	Chr2	178678490	Snv	G	C	rs35112591	Low	0.095	0.138	0.183	0.445	0.158	0.285
TTN	Chr2	178768917	Snv	T	C	rs4894045	Low	0.405	0.076	0.154	0.021	0.035	0.175
TTN	Chr2	178570835	Snv	C	T	rs1354658470	Low	0.000	-	-	-	-	-
TTN	Chr2	178531231	Snv	G	A	rs3813250	Low	0.523	0.432	0.595	0.288	0.744	0.448
TTN	Chr2	178741116	Snv	A	G	rs55895721	Low	0.000	0.088	0.006	-	-	-
TP53	Chr17	7675954	Snv	G	A	rs926582621	Modifier	0.000	-	-	-	-	-
TP53	Chr17	7676325	Indel	G	T	rs1376609066<br>rs59758982	Modifier	1.00	0.482	0.39	0.318	0.378	0.472
TP53	Chr17	7676301	Snv	C	G	rs17883323	Modifier	0.156	0.116	0.053	0.073	0.058	0.069
TP53	Chr17	7676483	Snv	C	T	rs1642785	Modifier	0.563	0.508	0.307	0.418	0.288	0.495
TP53	Chr17	7674797	Snv	A	G	rs1625895	Modifier	0.000	0.315	0.091	0.024	0.143	0.189
TP53	Chr17	7675361	Snv	T	C	rs9895829	Modifier	0.016	0.115	0.053	0.075	0.059	0.084
TP53	Chr17	7675327	Snv	A	C	rs2909430	Modifier	1.00	0.286	0.088	0.024	0.142	0.185
TP53	Chr17	7673642	Snv	C	T	rs966675626	Modifier	0.000	-	-	-	-	-
TP53	Chr17	7676154	Snv	G	C	rs1042522	Moderate	0.5859	0.669	0.317	0.414	0.285	0.492
TP53	Chr17	68819324	Indel	#	C	rs35117667	Low	0.016	0.029	-	-	-	-
PRKAA1	Chr5	40764806	Snv	A	C	rs930517925	Moderate	0	-	-	-	-	-
PSCA	Chr8	142681549	Snv	T	G	rs3736003	Modifier	0.027	0.179	0.020	0.115	0.014	0.041
PSCA	Chr8	142682339	Snv	C	G	rs1045547	Modifier	0.541	0.355	0.507	0.341	0.447	0.407
PSCA	Chr8	142682200	Snv	G	A	rs2976393	Modifier	0.813	0.355	0.509	0.341	0.448	0.407
PSCA	Chr8	142682583	Snv	G	A	rs2976396	Modifier	0.784	0.354	0.507	0.341	0.447	0.407
PSCA	Chr8	142682540	Snv	C	T	rs1045574	Modifier	0.789	0.354	0.507	0.342	0.447	0.407
PSCA	Chr8	142682204	Snv	C	G	rs2976394	Modifier	0.813	0.354	0.507	0.340	0.447	0.407
PSCA	Chr8	142682683	Snv	G	A	rs1045605	Modifier	0.700	0.354	0.507	0.341	0.447	0.408
PSCA	Chr8	142682272	Snv	C	A	rs10216533	Modifier	0.813	0.361	0.507	0.341	0.447	0.407
PSCA	Chr8	142681514	Snv	C	A	rs2976392	Modifier	0.265	0.368	0.507	0.341	0.446	0.407
PSCA	Chr8	142681306	Snv	C	T	rs2976391	Modifier	0.015	0.421	0.432	0.332	0.472	0.363
PSCA	Chr8	142680513	Snv	T	C	rs2294008	Modifier	0.813	0.368	0.504	0.342	0.447	0.407
PSCA	Chr8	142682332	Snv	C	G	rs2976395	Modifier	0.543	0.355	0.507	0.341	0.447	0.407

PSCA	Chr8	40764806	Snv	A	C	rs930517925	Moderate	0	-	-	-	-	-
PSCA	Chr8	142682072	Snv	G	A	rs2978982	Low	0.813	0.368	0.507	0.341	0.447	0.407
PSCA	Chr8	142682129	Snv	G	A	rs1045531	Low	0.4460	0.354	0.507	0.342	0.447	0.407
CDH1	Chr16	68737644	Snv	G	A	rs3743675	Modifier	0.500	0.363	0.223	0.23	0.119	0.207
CDH1	Chr16	68819472	Snv	C	T	rs35667437	Modifier	0.060	0.002	0.026	0.042	-	0.001
CDH1	Chr16	68737646	Indel	C	CA	.	Modifier	0.000	-	-	-	-	-
CDH1	Chr16	7676325	Indel	C	C	rs1370519975<br>rs34939176	Modifier	0.000	0.002	0.072	0.07	0.044	0.064
CDH1	Chr16	68737515	Indel	C	CGCCCCA<br>GCCCCGT	rs147838237<br>rs45625236	Modifier	0.079	-	-	-	-	-
CDH1	Chr16	68822138	Snv	G	A	rs33935154	Moderate	0.0833	0.051	0.004	-	0.001	-
CDH1	Chr16	68819324	Snv	C	T	rs730881667	Moderate	0.0833	-	-	-	-	-
CDH1	Chr16	68823538	Snv	C	T	rs1801552	Low	0.664	0.062	0.415	0.355	0.355	0.331
CDH1	Chr16	68828262	Snv	T	C	rs33964119	Low	0.000	0.058	0.059	0.069	0.035	0.044
CDH1	Chr16	68737469	Indel	T	TCC	rs3743674	Low	0.708	0.36	0.223	0.231	0.119	0.206

(-) No annotation; (*) Minor allele frequencies; (#) TTTTCCTCTTCAGGAGCAA; INDG: Indigenous Amazonian population, AFR: African population, AMR: American population, EAS: East Asian population, EUR: European population, SAS: South Asian population.

Table S2. Pairwise comparison (*p-value*) to significative results of allelic frequencies in Indigenous Amazonian population (INDG) and continental population (African (AFR), American population (AMR), East Asian (EAS), European (EUR), and South Asian (SAS)) described in the 1000 genomes database.

Gene	SNP Id	Var Type	Impact	INDG vs AFR*	INDG vs AMR*	INDG vs EAS*	INDG vs EUR*	INDG vs SAS*
TTN	rs7590037	Snv	Modifier	**0.0023**	**0.0053**	0	0	**0.0267**
TTN	rs397517782	Indel	Modifier	**0.0027**	**0.0287**	-	**0.0278**	**0.0051**
TTN	rs16866538	Snv	Moderate	0.2247	1	**0.0288**	1	**0.0748**
TTN	rs2042995	Snv	Moderate	1	1	1	**0.0023**	1
TTN	rs72646841	Snv	Modifier	**0.0257**	1	**0.0015**	1	1
TTN	rs1883085	Snv	Moderate	0	**0.001**	**0.0043**	0	0
TTN	rs2288327	Snv	Modifier	0	**0.0055**	0	**0.0375**	0
TTN	rs2562829	Snv	Moderate	0	**0.001**	**0.0043**	0	0
TTN	rs744426	Snv	Moderate	1	1	0	1	**0.0168**
TTN	rs3731749	Snv	Moderate	1	1	0	1	**0.0102**
TTN	rs4893852	Snv	Moderate	**0.0389**	0	0	0	**0.0011**
TTN	rs16866378	Snv	Moderate	**0.0257**	1	**0.0027**	1	1
TTN	rs17355446	Snv	Moderate	**0.0286**	1	**0.0778**	1	1
TTN	rs17076	Snv	Modifier	**0.0028**	0	0	1	**0.0101**
TTN	rs72650039	Snv	Modifier	**0.0092**	**0.0484**	-	-	-
TTN	rs397517532	Indel	Modifier	0	**0.0087**	1	1	**0.001**
TTN	rs3813243	Snv	Moderate	0.7531	1	**0.0027**	1	1
TTN	rs6723526	Snv	Moderate	**0.0480**	1	**0.0771**	1	1
TTN	rs11896637	Snv	Moderate	**0.0723**	1	-	1	-
TTN	rs72648270	Snv	Moderate	1	1	**0.0126**	1	1
TTN	rs747122	Snv	Moderate	1	**0.0057**	**0.0041**	0	**0.0191**
TTN	rs73038324	Snv	Moderate	**0.0257**	1	-	1	-
TTN	rs13421990	Snv	Modifier	0	1	0	**0.0127**	1
TTN	rs10203085	Snv	Modifier	1	0.1933	1	1	**0.0213**
TTN	rs34819099	Snv	Moderate	-	**0.048**	**0.01**	0.765	**0.011**

TTN	rs66839174	Snv	Moderate	**0.245**	**0.048**	-	-	-
TTN	rs3754949	Snv	Modifier	**0.091**	1	1	1	1
TTN	rs35813871	Snv	Moderate	1	1	**0.01**	**0.872**	1
TTN	rs13398235	Snv	Moderate	**0.002**	**0.002**	0	**0.0412**	**0.01**
TTN	rs397517532	Indel	Modifier	**0.001**	**0.074**	1	1	**0.013**
TTN	rs16866412	Snv	Moderate	0.7485	1	**0.008**	1	1
TTN	rs2303539	Snv	Modifier	0.275	1	0	1	**0.0178**
TTN	rs4894028	Snv	Moderate	0	0	0	0	**0.0204**
TTN	rs2303833	Snv	Modifier	**0.072**	1	**0.008**	1	1
TTN	rs72629779	Snv	Moderate	**0.0171**	1	-	-	-
TTN	rs3731746	Snv	Moderate	**0.007**	1	0	1	**0.043**
TTN	rs62178978	Snv	Modifier	1	1	**0.001**	1	1
TTN	rs12464703	Snv	Modifier	0	**0.062**	0.202	0	**0.0686**
TTN	rs55742743	Snv	Moderate	-	**0.0484**	-	1	0.0113
TTN	rs66677602	Snv	Modifier	0	**0.001**	**0.006**	0	0
TTN	rs2742351	Snv	Modifier	1	**0.003**	1	0.334	1
TTN	rs2306636	Snv	Moderate	0	**0.001**	**0.004**	0	0
TTN	rs72648915	Snv	Modifier	**0.016**	1	-	-	-
TTN	rs2042996	Snv	Moderate	1	1	**0.0294**	**0.0184**	1
TTN	rs73038323	Snv	Modifier	**0.026**	1	-	1	-
TTN	rs7585334	Snv	Moderate	0	1	**0.021**	0	**0.0174**
TTN	rs35147775<br>rs72394294	Indel	Modifier	**0.0154**	**0.009**	1	1	0
TTN	rs2303838	Snv	Moderate	**0.026**	1	0	1	**0.033**
TTN	rs72648982	Snv	Moderate	**0.003**	1	**0.001**	1	1
TTN	rs62621236	Snv	Moderate	0	**0.004**	**0.0394**	0	**0.044**
TTN	rs10183361	Snv	Modifier	**0.04**	0.119	1	1	**0.02**
TTN	rs72648911	Snv	Modifier	**0.002**	1	-	-	-
TTN	rs16866391	Snv	Moderate	0	1	1	-	**0.0051**
TTN	rs12476289	Snv	Moderate	0	**0.001**	**0.0037**	0	0

TTN	rs10164753	Snv	Moderate	0	**0.002**	**0.0394**	0	**0.044**
TTN	rs72646881	Snv	Moderate	**0.0421**	1	-	1	-
TTN	rs56142888	Snv	Moderate	0	**0.019**	**0.0632**	0	0
TTN	rs16866416	Snv	Modifier	1	1	0	**0.002**	1
TTN	rs79466278	Snv	Moderate	0.245	**0.0484**	-	-	-
TTN	rs2291313	Snv	Modifier	0	0	**0.0044**	0	0
TTN	rs9808377	Snv	Moderate	1	1	0.172	**0.0028**	1
TTN	rs3829747	Snv	Moderate	1	1	0	1	**0.0168**
TTN	rs890578	Snv	Modifier	0	**0.006**	0	**0.0363**	0
TTN	rs7606485	Snv	Modifier	0	**0.004**	0.132	0	**0.047**
TTN	rs3829746	Snv	Low	1	1	**0.035**	**0.0219**	1
TTN	rs10497520	Snv	Moderate	**0.005**	0	0	1	0
TTN	rs11888217	Snv	Moderate	0	**0.004**	0.132	0	**0.063**
TTN	rs4894048	Snv	Moderate	0	**0.0124**	0	0	**0.005**
TTN	rs4893853	Snv	Moderate	0	**0.002**	0	0	**0.006**
TTN	rs922984	Snv	Modifier	0	**0.049**	1	1	1
PSCA	rs2976393	Snv	Modifier	0	**0.001**	0	0	0
PSCA	rs2976396	Snv	Modifier	0	**0.0065**	0	0	0
PSCA	rs1045574	Snv	Modifier	0	**0.0065**	0	0	0
PSCA	rs1045605	Snv	Modifier	0	0.471	0	**0.019**	**0.001**
PSCA	rs2976392	Snv	Modifier	1	**0.04**	1	0.801	1
CDH1	rs33935154	Snv	Moderate	1	**0.048**	-	**0.01**	-
CDH1	rs3743675	Snv	Modifier	1	**0.001**	**0.001**	0	0
CDH1	rs35667437	Snv	Modifier	**0.0286**	1	1	-	**0.018**
TP53	rs1042522	Snv	Moderate	**0.006**	1	1	1	1
TP53	rs1642785	Snv	Modifier	1	**0.02**	1	**0.003**	1
TP53	rs1625895	Snv	Modifier	0	0.557	1	**0.0215**	**0.001**

(-) No annotation; *p-value obtained by Fisher's exact test; bold: significant result (p-value ≤ 0.05).

Table S3. List of abreviations and acronyms.

Abreviation	Content
GC	Gastric Cancer
CDH1	Cadherin-1
TP53	Protein Tumor
PSCA	Prostate Stem Cell Antigen
PRKAA1	Protein Kinase AMP-Activated Catalytic Subunit Alpha 1
TTN	Titina
SNV	Single Nucleotide Variant
SNP	Single Nucleotide Polimorphism
INDG	Indigenous population
AFR	African population
AMR	American population
EAS	East Asian population
EUR	European population
SAS	South Asian populations
Chr	Chromossome
MDS	Multidimensional Scaling Plot
IBGE	Brazilian Institute of Geography and Statics
